# Transcriptome analysis revealed the expression levels of genes related to abscisic acid and auxin biosynthesis in grapevine (*Vitis vinifera* L.) under root restriction

**DOI:** 10.3389/fpls.2022.959693

**Published:** 2022-08-24

**Authors:** Lei Wang, Hui Li, Jiajia Li, Guanhan Li, Muhammad Salman Zahid, Dongmei Li, Chao Ma, Wenping Xu, Shiren Song, Xiangyi Li, Shiping Wang

**Affiliations:** Department of Plant Science, School of Agriculture and Biology, Shanghai Jiao Tong University, Shanghai, China

**Keywords:** *Vitis vinifera* L., root restriction, abscisic acid, auxin, root system, high-throughput sequencing

## Abstract

The root system is essential for the stable growth of plants. Roots help anchor plants in the soil and play a crucial role in water uptake, mineral nutrient absorption and endogenous phytohormone formation. Root-restriction (RR) cultivation, a powerful technique, confines plant roots to a specific soil space. In the present study, roots of one-year-old “Muscat Hamburg” grapevine under RR and control (nR) treatments harvested at 70 and 125 days after planting were used for transcriptome sequencing, and in total, 2031 (nR7 vs. nR12), 1445 (RR7 vs. RR12), 1532 (nR7 vs. RR7), and 2799 (nR12 vs. RR12) differentially expressed genes (DEGs) were identified. Gene Ontology (GO) enrichment analysis demonstrated that there were several genes involved in the response to different phytohormones, including abscisic acid (ABA), auxin (IAA), ethylene (ETH), gibberellins (GAs), and cytokinins (CTKs). Among them, multiple genes, such as PIN2 and ERF113, are involved in regulating vital plant movements by various phytohormone pathways. Moreover, following RR cultivation, DEGs were enriched in the biological processes of plant-type secondary cell wall biosynthesis, the defense response, programmed cell death involved in cell development, and the oxalate metabolic process. Furthermore, through a combined analysis of the transcriptome and previously published microRNA (miRNA) sequencing results, we found that multiple differentially expressed miRNAs (DEMs) and DEG combinations in different comparison groups exhibited opposite trends, indicating that the expression levels of miRNAs and their target genes were negatively correlated. Furthermore, RR treatment indeed significantly increased the ABA content at 125 days after planting and significantly decreased the IAA content at 70 days after planting. Under RR cultivation, most ABA biosynthesis-related genes were upregulated, while most IAA biosynthesis-related genes were downregulated. These findings lay a solid foundation for further establishing the network through which miRNAs regulate grapevine root development through target genes and for further exploring the molecular mechanism through which endogenous ABA and IAA regulate root architecture development in grapevine.

## Introduction

Roots, which are the most crucial parts of plants, collectively form a root system, which is vital for growth and for anchoring plants into the soil and plays a pivotal role in water and mineral nutrient absorption as well as the formation of endogenous phytohormones (Tardieu, 1996; Hopmans and Bristow, 2002). Grapevine roots, which are fleshy, are crucial for storing essential nutrients. Thus, grapevine farmers continuously seek strategies to keep the root system healthy (Richards, 1983). Grapevine is propagated via cuttings because they are easily obtained, and roots quickly emerge from cuttings and maintain the characteristics of the female parent (Smart et al., 2006). Previous research indicated that the roots produced by grapevine cuttings can be classified into five stages based on the various development stages: initial cultivation (stage I), preliminary development (stage II), even change (stage III), root system formation (stage IV), and root architecture stability (stage V) (Li et al., 2020a). Other studies have demonstrated that multiple environmental factors, including soil substrate selection, nutrition, and irrigation, have an important role in fostering root vigor and vitality (Cornish et al., 1984; Van Zyl, 1984).

Root restriction (RR) cultivation is an agronomic technique that has been largely developed in recent years. It involves the use of some physical or ecological methods to control the range of plant root systems in a particular soil space as well as the regulation of vegetative and reproductive growth through the regulation of the development of roots for obtaining high yields and high efficiency in grapevine production (Wang et al., 2001, 2012; Yu et al., 2015). For example, the total sugar content of grape juice as well as grape berries under RR cultivation was found to be higher than that of control fruits, and acid invertase was the most critical enzyme that induces sugar accumulation (Xie et al., 2009). Duan et al. (2019) demonstrated that RR significantly affected the metabolome of berry pericarp tissue, especially the accumulation of anthocyanins before the veraison stage, accompanied by advanced veraison stage of grape berries. It has been reported that the grapevine root architecture under RR cultivation is different from that under standard cultivation; the root system displayed an increase in adventitious and lateral root numbers and root tip degeneration after RR cultivation. Moreover, the roots exhibited spiral curling, which contributed to an accelerated root metabolic rate; the ability of roots to absorb nutrients increased, which directly led to a significant improvement of fruit quality (Li et al., 2020b). During the growth of grapevine, the content of endogenous phytohormones changes significantly, along with the development of phenotypes and the improvement of fruit quality. Interestingly, it has been confirmed that the formation of several phenotypic parameters (sugar accumulation, pericarp coloration, and root architecture improvement) under RR cultivation is affected by endogenous ABA and IAA levels. Guo et al. (2014) reported that the ABA contents in the roots, stems, leaves and berries of grapevine under RR cultivation were significantly higher than those of the control group, while the IAA contents in roots, stems and leaves of grapevine under RR cultivation were significantly lower than those of the control group (Leng et al., 2018; Li et al., 2022c). Further investigation at the molecular level verified that 12 DEGs involved in ABA biosynthetic or catabolic pathways, including *crtZ*, *ZEP/ABA1*, *NCED*, *ABA2*, *AAO3* and *CYP707A*, were identified under RR cultivation, which strongly indicated that root restriction had the greatest impact on the biosynthesis and catabolism of ABA and ultimately regulated the occurrence of a series of biological activities in grapevine plant by mediating the formation of ABA (Li D. et al., 2021). In addition, RR cultivation increased the ABA content in “Muscat Hamburg” grape berries and significantly increased the expression level of the ABA response factor VvGRIP55 in fruits at the early veraison stage, veraison stage and maturity stage. The binding of VvGRIP55 to the promoter of the transcription factor VvMYB15 transcription factor regulates sugar accumulation and activates *VvMYB15* expression, and VvMYB15 transcription factor further binds to the promoter of the sugar transport gene VvSWEET10 and activates its expression, ultimately promoting sugar accumulation in grape berries (Li et al., 2022a). Moreover, both RR-inducible transcription factors (VvMYB15 transcription factor and VvWRKY40 transcription factor) specifically bind to the promoter regions of *VvF3′5′H* and *VvUFGT*, confirming that VvMYB15 transcription factor and VvWRKY40 transcription factor synergistically promote anthocyanin accumulation and increase the expression levels of anthocyanin biosynthesis-related genes (Li et al., 2022b). Because RR mainly restricts the growth of vine roots by physical means, the change in root configuration under RR cultivation has also been the focus of research. However, the relationship between the variation in ABA or IAA levels under RR cultivation and the differences in the grape root system remain unknown.

In recent years, sequencing technology has developed quickly and has played an essential role in studying biological issues (Slatko et al., 2018). Generally, the transcriptome refers to the collection of all the transcripts, including mRNA, rRNA, tRNA, and non-coding RNA, in a cell under a particular physiological condition (Longo et al., 2021). Transcriptomics can be used to study gene function and structure at the whole-plant level to study specific biological processes. Transcriptomics has been widely used in plant candidate gene discovery, functional identification, and genetic improvement. RNA sequencing (RNA-seq) technology has become a vital means of transcriptome research (An et al., 2018). At present, RNA-seq has been extensively applied to model plant species such as maize (*Zea mays*), *Arabidopsis thaliana*, rice (*Oryza sativa*), and tomato (*Solanum lycopersicum*) (Zhang et al., 2010; Hansey et al., 2012; Zhu et al., 2015; Klepikova et al., 2016). High-throughput sequencing technology has revolutionized the sequencing process in plant science. Specifically, transcriptome sequencing was performed on grape berries and roots after plastic film mulching and grass cover, the findings of which revealed changes in tannin, anthocyanin, soluble solids, and titratable acid contents in the berries (Mir et al., 2019). These changes may be related to high-affinity nitrate transporter 2.1 (NRT2.1), NRT2.4, and glutamine synthetase expression detected in the roots, indicating that mulching could improve fruit quality by activating nitrogen transport (Wang et al., 2020). In addition, the primary mechanism of calcium-induced anthocyanin biosynthesis revealed by transcriptome sequencing and the findings associated with DEGs show that this mechanism might affect grape pericarp coloring in multiple calcium transport and signal transduction pathways. Validating transcriptome sequencing is an indispensable step in revealing the improvement of plant phenotypes (Yu et al., 2020). The above findings provide ideas and directions for further research on combining transcriptome data to elucidate molecular mechanisms to illustrate the development of different grapevine organs. However, unfortunately, research on further revealing the changes in root architecture under RR cultivation by transcriptome sequencing has not yet been carried out.

In this study, a transcriptome sequencing-based study of gene expression variation in grapevine roots during conventional and RR cultivation was implemented. The mechanism through which miRNAs act on target genes during grapevine root development was examined, followed by a transcriptome sequencing correlation analysis with published miRNA sequencing data, aiming to determine the synergistic/antagonistic effects of transcriptional regulation and posttranscriptional regulation on root development in grapevine. Additionally, the contents of various endogenous phytohormones and the expression levels of ABA and IAA biosynthesis- and catabolism-related genes under the two cultivation modes were also measured to validate our previous hypothesis that endogenous ABA and IAA play vital roles in the changes in grape root architecture and to provide reliable reference data for prospective studies on the molecular mechanisms governing variations in root architecture regulated by endogenous ABA and IAA under RR cultivation.

## Materials and methods

### Plant materials and experimental design

A 1-year-old grapevine (*Vitis vinifera* L. cv. “Muscat Hamburg”) cuttings were planted in the greenhouse of the Fruit Tree Laboratory at Shanghai Jiao Tong University (31°110 N, 121°290 W) in 2017. All the cuttings were planted in a mixture of substrate soil, organic fertilizer, and perlite (1:1:1). The roots were restricted to a container with a diameter of 30 cm and a height of 30 cm; this container was separated from the ground by a tray during RR cultivation. Under regular cultivation (referred to as nR), the cuttings were cultivated on the ground in 40 cm tall beds consisting of mixed substrate. The aboveground management and irrigation practices were the same. All of treatments involved single-vine growth with no topping. Then, 24 grapevine root samples from the two different cultivation treatments were collected at 12 time points (10, 20, 30, 40, 50, 60, 70, 80, 90, 100, 110, and 125 days after planting). According to previous root morphology observations, the root architecture differed beginning 70 days after planting (DAP) under root restriction cultivation.

### Transcriptome sequencing library construction

The same grapevine root samples previously harvested for miRNA sequencing (sampled at 70 and 125 days after planting) under RR and nR cultivation were used for transcriptome sequencing. These four root samples were named nR7, nR12, RR7, and RR12, as shown in Supplementary Figure 1. A total of three biological replicates were evaluated, recorded as A, B, and C. The sequencing was performed by staff at Shanghai OE Biomedical Technology Co., Ltd. (SHN, China).

According to the manufacturer’s instructions, total RNA was extracted from the roots using the CTAB method (Gambino et al., 2008). The concentration and purity of the RNA were detected with a NanoDrop 2000 (Thermo Fisher Scientific, United States) and an Agilent 2100 Bioanalyzer (Agilent Technologies, United States). Magnetic beads with Oligo (dT) were used for mRNA enrichment and were broken into short fragments. The fragmented mRNAs were reverse transcribed with six-base random primers to generate single-stranded cDNA, after which double-stranded cDNA was synthesized. After a purification process, the double-stranded cDNA was subjected to PCR amplification, polyadenylated, and ligated to sequencing adapters. The length and quality of the library were determined by an Agilent 2100 Bioanalyzer and sequenced using an Illumina HiSeq™ 2500. The raw RNA sequencing data have been uploaded and deposited into the National Center for Biotechnology Information (NCBI) BioProject database under accession number PRJNA808789.

### Analysis of protein-coding gene expression

The raw sequencing data were preprocessed by Trimmomatic software (Bolger et al., 2014). The quality control steps included (1) removing the adaptors; (2) removing low-quality reads; and (3) removing low-quality bases from the 3′ and 5′ ends and obtaining clean reads. The clean read sequences were aligned to the grapevine *Pinot Noir* genome sequence^1^, and the number of mapped reads as well as the position information of the reads aligned to the genome was recorded. HTSeq-count software was used to compare the number of reads with the number of protein-coding genes in each sample (Anders et al., 2015), and cufflinks software was used to calculate the FPKM (fragments per kilobase per million reads) value of the protein-coding genes (Roberts et al., 2011). The calculation formula for FPKM is FPKM(A) = number of fragments aligned to gene A/number of components aligned to all genes × length of gene (A) × 10^9^.

### Analysis of differentially expressed genes

According to the calculated FPKM values, the genes differentially expressed between different samples were analyzed. The primary screening steps were as follows: (1) DESeq software was used to standardize the number of gene counts in each sample (finally, the basal value was used to estimate the expression level) (Anders and Huber, 2012), and the different expression fold-changes were calculated. (2) A negative binomial distribution test was performed to determine the significance of the different readings. Two criteria were used to determine the same gene’s differential expression between comparison samples. One is based on the expression fold-change, which is the fold-change of the expression level of the same gene between two samples. The other is based on the FDR value, which was calculated by first calculating the p value of each gene and then using the FDR error control method to correct the p value for multiple hypothesis testing. The criteria for the final screened DEGs were *p* < 0.05 and an expression fold-change greater than 2. GO (Gene Ontology) enrichment analysis was performed after obtaining the DEGs. The GO entries for which the number of DEGs was greater than 2 in the classification categories of biological function, cell composition, and molecular function were screened (Alexa and Rahnenführer, 2009).

### MicroRNA target gene prediction

The TargetFinder website was used to predict the target genes of differentially expressed miRNAs using previously available miRNA sequencing data collected from grapevine under root restriction (accession number: PRJNA601829) (Bo and Wang, 2005). Moreover, we compared the predicted target genes with the DEGs in the transcriptome data to identify the combinations of differentially expressed miRNAs and target genes.

### Extraction and determination of endogenous phytohormone contents

Abscisic acid, IAA, GA, methyl jasmonate (MeJA), brassinolide (BR), and zeatin riboside (ZR) were extracted according to our published methods, with minor modifications (Li J. et al., 2021). Grape berries without seeds were crushed into a powder in liquid nitrogen, and 50 mg of the powder and an extraction solution (80% methanol, v/v) were mixed together. After centrifuging at 10,000 rpm for 20 min, the supernatant was eluted through a SepPak C18 cartridge (Waters, MA, United States) to remove polar compounds. Finally, 0.5 mL extracting solution containing ABA, IAA, GA, MeJA, BR, and ZR were obtained, and three biological replicates were included.

A Poroshell EC-120 (3 μm × 4.6 mm × 100 mm) C18 column (Waters, MA, United States) installed in UPLC-HRMS system (Waters, MA, United States), was used for compound separation. The elution solutions included solvent A (0.1% acetic acid in water) and solvent B (0.05% acetic acid in acetonitrile). The flow rate in the UPLC-HRMS system was set to 0.3 mL/min. The gradient elution program was as follows: 0–6.25 min, 10% B; 6.25–7.5 min, 40% B; 7.5–10.6 min, 90% B; and 10.6–13.5 min, 10% B. Next, after measured the peak area of standards (ABA, IAA, GA, MeJA, BR, ZR with suitable concentration gradient) in the same UPLC-HRMS system, the standard curves of each phytohormone were obtained accordingly. In terms of above standard curves, the specific content of each phytohormone in treatment groups were finally conversed.

### qRT-PCR analysis

The extraction of total RNA in the grape roots of the different treatments was carried out on an RNA Prep Pure Plant Plus Kit (TaKaRa, Dalian, China). A BIO-RADXR gel imaging analysis system (Bio-Rad, CA, United States) was used to determine the purity and integrity of the RNA extracted. One microgram of total RNA was extracted by the use of a PrimeScript™ RT Reagent Kit in combination with gDNA Eraser (Perfect Real Time) (TaKaRa, Dalian, China) according to the manufacturers’ instructions. Then, first-strand cDNA was obtained.

The total final volume was 10 μl, consisting of 1 μl of cDNA, 5 μl of TB Green^®^ Fast qPCR Mix, 3 μl of ddH_2_O, and 1 μl of a forward and reverse primer mixture. Then, qRT-PCR was implemented in a CFX connect Real Time PCR Detection System (Bio-Rad, CA, United States), and the sequences of all the genes and transcription factors identified in this study were acquired from the EnsemblPlants database^2^. The qPrimerDB-qPCR Primer Database^3^ was used to design the primers used in this study. The specific information of all the primer sequences used is shown in Supplementary Table 1, and the reactions were carried out as follows: 95°C for 20 s, followed by 39 cycles of 95°C for 15 s, 55°C for 15 s and 60°C for 15 s. The 2^–ΔΔCt^ method was used for the determination of relative gene expression.

### Data analysis and figure construction

To assess the significant differences in different phytohormones contents between the nR cultivation and RR cultivation treatments, the independent sample T-test method of SPSS 16.0 (IBM, Armonk, NY, United States) was carried out, specifically, selected the test variables (phytohormone contents) and grouping variables (nR and RR), set the group values, and judged the significance according to the test results of variance homogeneity (if *p* < 0.05, indicated as *; if *p* < 0.01, indicated as ^**^). GO enrichment and KEGG pathway enrichment analysis of DEGs were performed respectively using R based on the hypergeometric distribution (Kanehisa et al., 2007). A heatmap was also constructed to visualize the results of a hierarchical cluster analysis. Figures related to quantification of phytohormones were constructed by GraphPad Prism 9.0 (GraphPad Software, Inc., San Diego, CA, United States) and Visio 2020 (Microsoft, SEA, United States). Figures such as GO enrichment and cluster analysis were exported from R.

## Results

### Transcriptome sequencing data from different root samples

The 12 sequencing samples generated a total of 85.72 GB of clean data. The raw data ranged from 5.40 to 8.07 GB in each instance, while the clean reads ranged from 44.09 to 55.47 MB. The Q30 distribution was between 92.41% and 93.40%, and the average GC content was 45.10%. In total, 90.59 to 91.80% of the clean reads were mapped to the grapevine (Table 1).

**TABLE 1:** The statistical information of transcriptome sequencing.

Sample	raw_reads	clean_reads	raw_bases	clean_bases	valid_bases	Q30	GC	Total mapped reads
nR7A	47.59M	45.80M	7.14G	6.65G	0.9318	0.9292	0.46	42,018,035 (91.73%)
nR7B	52.83M	51.02M	7.92G	7.41G	0.9351	0.9332	0.4604	46,832,875 (91.80%)
nR7C	46.47M	44.55M	6.97G	6.45G	0.9247	0.9253	0.46	40,805,291 (91.59%)
RR7A	49.53M	47.69M	7.43G	6.93G	0.9324	0.9306	0.4611	43,592,878 (91.41%)
RR7B	45.73M	44.09M	6.86G	6.40G	0.9334	0.9314	0.4614	40,288,707 (91.39%)
RR7C	56.26M	54.31M	8.44G	7.89G	0.935	0.934	0.4607	49,670,424 (91.45%)
nR12A	48.15M	46.57M	7.22G	6.76G	0.9354	0.9337	0.4617	42,201,039 (90.61%)
nR12B	49.11M	47.49M	7.37G	6.89G	0.9348	0.9336	0.4622	43,060,329 (90.67%)
nR12C	51.35M	49.46M	7.70G	7.18G	0.9324	0.93	0.4617	44,805,942 (90.59%)
RR12A	57.73M	55.47M	8.66G	8.07G	0.9325	0.9282	0.461	50,508,974 (91.06%)
RR12B	54.94M	52.57M	8.24G	7.65G	0.9278	0.9241	0.4609	47,849,577 (91.02%)
RR12C	53.09M	51.15M	7.96G	7.44G	0.9343	0.9307	0.4603	46,597,472 (91.11%)

**FIGURE 1:**
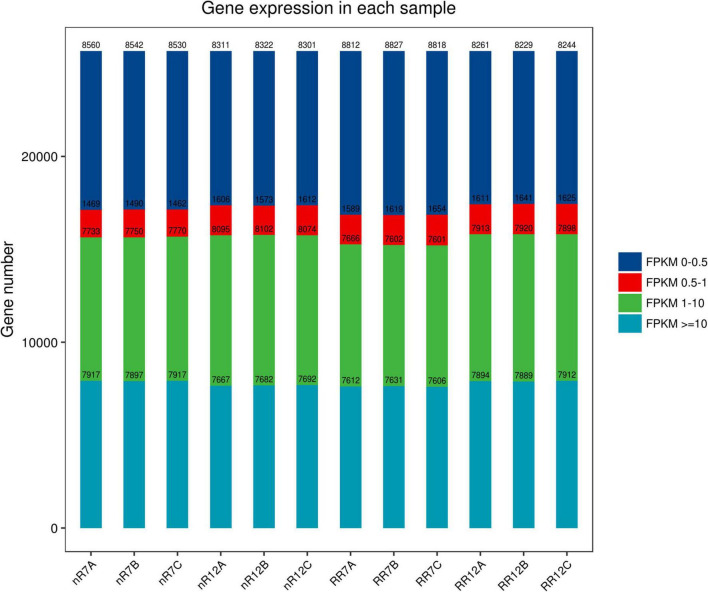
The distribution of FPKM expression in nR7, RR7, nR12, and RR12 represent at the 70 DAP (days after planting days) and 125 DAP sampling points under nR and RR cultivation. A total of four FRKM were classified in this study, including FRKM 0–0.5, FRKM 0.5–1, FRKM 1–10, and FRKM ≥ 10.

**FIGURE 2:**
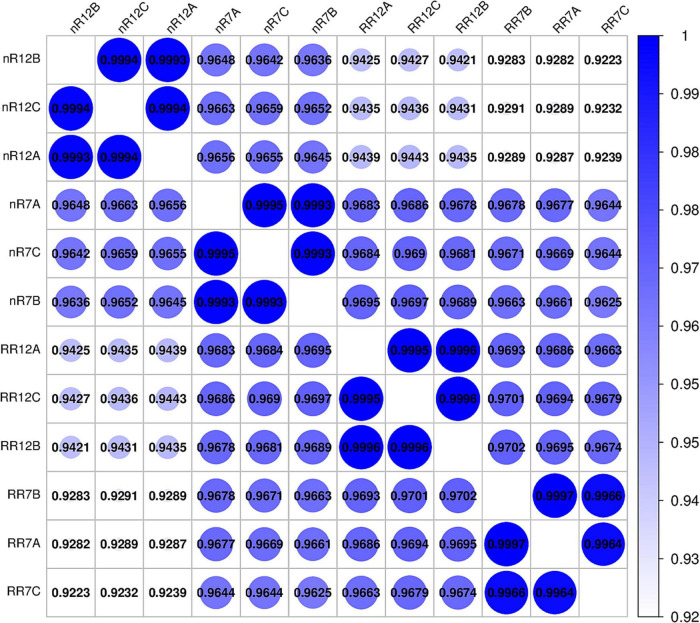
The heat map of the correlation coefficient between different samples, including nR7, RR7, nR12, and RR12 represents the 70 DAP and 125 DAP sampling points under nR and RR cultivation. The specific correlation value was reflected on the bubble.

The expression levels of each gene in the different samples showed that the FPKM values differed greatly. The FPKM values were divided into four intervals from 0 to 0.5 (Group A), 0.5−1 (Group B), 1−10 (Group C), and ≥10 (Group D). The results indicated that Group B contained the fewest genes (1462−1654 genes), while the number of genes in the other three groups showed no significant differences, each group of which had approximately 7000−8000 genes. In addition, the distribution of FPKM values among different cultivation treatments and different developmental stages was similar (Figure 1). In particular, analysis of gene expression levels between different samples revealed high correlation coefficients between replicates of the same root samples. Moreover, the correlation coefficients were also higher than 0.92 between different sequencing samples, which indicated that the repeatability between treatment groups was high, and the data associated with the nR and RR cultivation treatments were strongly correlated (Figure 2).

### Analysis of genes differentially expressed between different comparison groups

**FIGURE 3:**
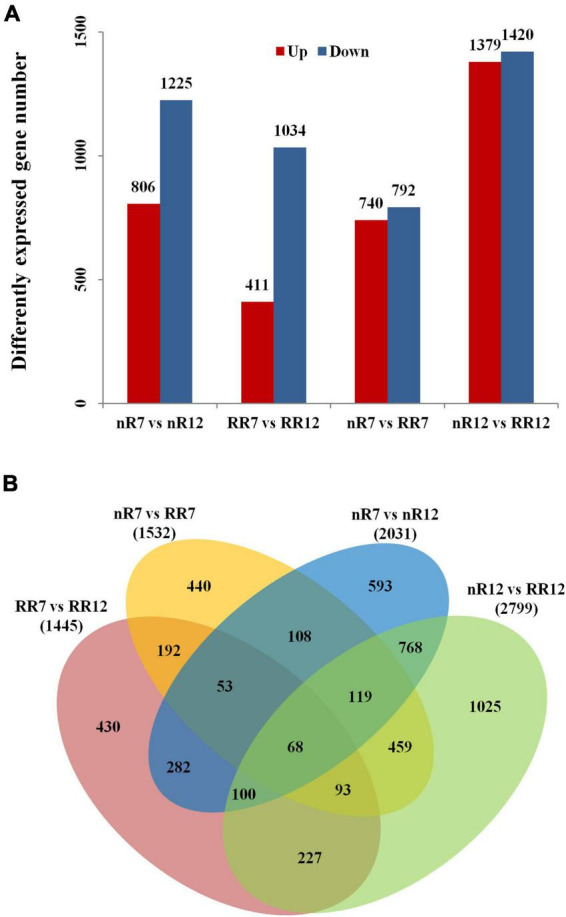
Differentially expressed gene number analysis. **(A)** Differentially expressed gene numbers between different comparison groups (nR7 vs. nR 12, RR7 vs. RR12, nR7 vs. RR7, and nR7 vs. RR12). The annotations of up and down mean the number of significantly up-regulated or down-regulated genes. **(B)** Mutual and unique differentially expressed genes between different comparison groups (nR7 vs. nR 12, RR7 vs. RR12, nR7 vs. RR7, and nR7 vs. RR12). nR7, RR7, nR12, and RR12 represent the 70 DAP and 125 DAP sampling points under nR and RR cultivation.

To further investigate the number of genes differentially expressed at different root development stages under RR cultivation and nR cultivation, we analyzed the DEGs between comparison groups. The results revealed 2,031 and 1,445 DEGs at different root developmental stages in the nR7 vs. nR12 and RR7 vs. RR12 comparison groups, respectively. The number of downregulated genes was much higher than that of upregulated genes—nearly twofold higher (Figure 3A). The number of upregulated and downregulated genes was similar in the different cultivation treatments. Moreover, the number of DEGs in the nR12 vs. RR12 comparison group was significantly higher than that of the nR7 vs. RR7 comparison group (Figure 3A).

To further investigate the number of common and unique DEGs under the different cultivation treatments, as shown in Figure 3B, 1532 and 2799 DEGs were obtained in the nR7 vs. RR7 and nR12 vs. RR12 comparison groups. There were 503 common DEGs in the nR7 vs. nR12 and RR7 vs. RR12 comparison groups at different root development stages. Moreover, 739 common DEGs were obtained in the nR7 vs. RR7 and nR12 vs. RR12 comparison groups after root restriction cultivation (Figure 3B).

### Gene ontology annotation analysis of genes differentially expressed between the different comparison groups

The expression of five endogenous phytohormone biosynthesis-related genes, including those involved in ABA, was visualized via a heatmap to further investigate the influence of nR and RR on endogenous phytohormone biosynthesis-related genes or transcription factors. The GO enrichment analysis showed several genes among the common DEGs that responded to other phytohormone processes. Each gene’s upregulation and downregulation patterns differed in the nR7 vs. RR7 and nR12 vs. RR12 comparison groups. Further classification analysis revealed that 14 DEGs responded to IAA, including the auxin-responsive proteins IAA3 and IAA29 and the PIN transporters PIN2 and PIN6. IAA3, IAA 29, and PIN6 showed an upregulation trend under nR treatment.

In contrast, PIN2 showed an upregulation trend under RR treatment. Moreover, 14 DEGs were found to respond to ABA, most of which were upregulated after RR cultivation. There were multiple senescence-specific SAG12 cysteine proteases, and only two DEGs (SUGTL2 and MFT) were downregulated; the others were all upregulated under the RR treatment. We also found that a total of 18 DEGs that responded to ETH, including multiple ethylene response factor (ERF) members that were downregulated under RR treatment, as well as MYB27 transcription factor and MYB48 transcription factor that were highly upregulated under RR treatment.

**FIGURE 4:**
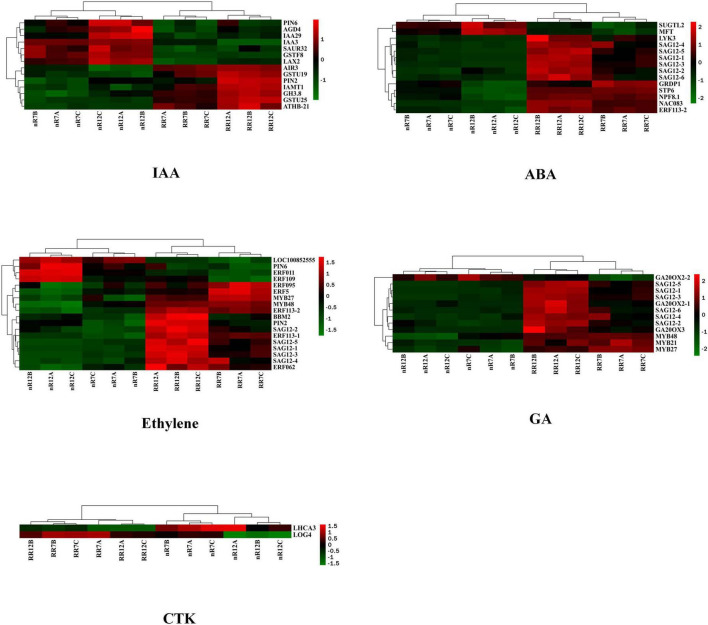
Heat map analysis of differentially expressed genes in response to phytohormones IAA, ABA, ETH, GA, and CTK. nR7, RR7, nR12, and RR12 represent the 70 DAP and 125 DAP sampling points under nR and RR cultivation.

Furthermore, 12 DEGs responded to GA and were mostly upregulated after RR cultivation, including the typical GA biosynthesis-related genes gibberellin 20 oxidase 2 (*GA20OX2*, *Vitvi04g01719*) and gibberellin 20 oxidase 3 (*GA20OX3*, *Vitvi02g01769*) and multiple genes encoding MYB transcription factors. Moreover, except for gibberellin 20 oxidase 2-2 (*GA20OX2-2*, *Vitvi16g00890*), the majority of the DEGs were upregulated during the RR treatment. For DEGs related to the CTK response, the results demonstrated that two genes respond to CTK, and cytokinin riboside 5′-monophosphate phosphoribohydrolase (*LOG4*, *LOC10025518*) was upregulated under RR cultivation. At the same time, photosystem I chlorophyll a/b-binding protein 3-1 (*LHCA3*, *LOC100232927*) was downregulated after RR cultivation. Overall, some genes, such as SAG12s, responded to multiple phytohormone processes, especially those involving GA, ETH, and ABA. In addition, auxin efflux carrier component 2 (*PIN2*, *LOC100256460*) and photosystem I chlorophyll a/b-binding protein 3-1 (*PIN6*, *LOC100250503*) responded to IAA and ETH, ethylene-responsive transcription factors (*ERF113*, *LOC100266913*) responded to ETH and ABA, and MYB59 transcription factors (MYB59, *LOC100260318*) and ODORANT1 proteins (MYB48, *LOC100263072*) responded to GA and ETH (Figure 4). More information about the DEGs is provided in Table 2.

**TABLE 2:** The annotation information of differentially expressed genes in response to plant hormones auxin (IAA), abscisic acid (ABA), ethylene (ETH), gibberellin (GA), brassinolide (BR), and cytokinins (CTKs) after root restriction cultivation.

ID	Gene	Product	Up_down regulation	GO_id	GO_term	Pathway	Pathway_description
GST1	GSTU25	glutathione S-transferase	Down	GO:0004364,GO:0009734	glutathione transferase activity| auxin-activated signaling pathway	ko00480,ko00980,ko00982	Glutathione metabolism| Metabolism of xenobiotics by cytochrome P450| Drug metabolism−cytochrome P450
LOC100259478	GSTU19	probable glutathione S-transferase	Down	GO:0004364,GO:0009734	glutathione transferase activity| auxin-activated signaling pathway	ko00480,ko00980,ko00982	Glutathione metabolism| Metabolism of xenobiotics by cytochrome P450| Drug metabolism−cytochrome P450
LOC100242734	GSTU19L	probable glutathione S-transferase	Down	GO:0004364,GO:0009734	glutathione transferase activity| auxin-activated signaling pathway	ko00480,ko00980,ko00982	Glutathione metabolism| Metabolism of xenobiotics by cytochrome P450| Drug metabolism−cytochrome P450
LOC100252749	GSTF8	glutathione S-transferase	Up	GO:0004364,GO:0009734	glutathione transferase activity| auxin-activated signaling pathway	ko00480,ko00980,ko00982	Glutathione metabolism| Metabolism of xenobiotics by cytochrome P450| Drug metabolism−cytochrome P450
LOC100256460	PIN2	auxin efflux carrier component 2	Down	GO:0009925,GO:0005783, GO:0016021,GO:0000323, GO:0005886,GO:0010329, GO:0010252,GO:0009926, GO:0009734,GO:0009958, GO:0009733,GO:0009723, GO:0009749	basal plasma membrane| endoplasmic reticulum| integral component of membrane| lytic vacuole| plasma membrane| auxin efflux transmembrane transporter activity| auxin homeostasis| auxin polar transport| auxin-activated signaling pathway| positive gravitropism| response to auxin| response to ethylene| response to glucose		
LOC100250503	PIN6	auxin efflux carrier component 6	Up	GO:0005783,GO:0005789, GO:0016021,GO:0005886, GO:0010329,GO:0010252, GO:0009926,GO:0009734, GO:0010540,GO:0010105, GO:1901332,GO:0048767	endoplasmic reticulum| endoplasmic reticulum membrane| integral component of membrane| plasma membrane| auxin efflux transmembrane transporter activity| auxin homeostasis| auxin polar transport| auxin-activated signaling pathway| basipetal auxin transport| negative regulation of ethylene-activated signaling pathway| negative regulation of lateral root development| root hair elongation		
LOC100253820	LAX2	auxin transporter-like protein 2	Up	GO:0016021,GO:0005886, GO:0015293,GO:0006865, GO:0009734	integral component of membrane| plasma membrane| symporter activity| amino acid transport| auxin-activated signaling pathway	ko04075	Plant hormone signal transduction
LOC100250868	GH3.8	probable indole-3-acetic acid-amido synthetase GH3.1	Down	GO:0016874,GO:0009733	ligase activity| response to auxin	ko04075	Plant hormone signal transduction
LOC100264878	IAA3	auxin-induced protein 22D	Up	GO:0005634,GO:0009734, GO:0006355,GO:0006351	nucleus| auxin-activated signaling pathway| regulation of transcription, DNA-templated| transcription, DNA-templated	ko04075	Plant hormone signal transduction
LOC100266398	IAA29	auxin-responsive protein IAA9,	Up	GO:0005634,GO:0003700, GO:0009734,GO:0009733, GO:0010218,GO:0010114, GO:0006351	nucleus| transcription factor activity, sequence-specific DNA binding| auxin-activated signaling pathway| response to auxin| response to far red light| response to red light| transcription, DNA-templated	ko04075	Plant hormone signal transduction
LOC100253069	ATHB-21	homeobox-leucine zipper protein ATHB-40	Down	GO:0005634,GO:0043565, GO:0003700,GO:0009733, GO:0006351	nucleus| sequence-specific DNA binding| transcription factor activity, sequence-specific DNA binding| response to auxin| transcription, DNA-templated		
LOC100254173	AGD4	ADP-ribosylation factor GTPase-activating protein AGD3	Up	GO:0005829,GO:0005886, GO:0030140,GO:0005096, GO:0046872,GO:0035091, GO:0006897,GO:0009965, GO:0010087,GO:0009733, GO:0010051	cytosol| plasma membrane| trans-Golgi network transport vesicle| GTPase activator activity| metal ion binding| phosphatidylinositol binding| endocytosis| leaf morphogenesis| phloem or xylem histogenesis| response to auxin| xylem and phloem pattern formation	ko04144	Endocytosis
LOC100255614	AIR3	subtilisin-like protease SBT5.3	Down	GO:0005618,GO:0005576, GO:0004252,GO:0010102, GO:0009733	cell wall| extracellular region| serine-type endopeptidase activity| lateral root morphogenesis| response to auxin		
LOC100855086	SAUR32	auxin-responsive protein SAUR50-like	Up	GO:0005737,GO:0009734, GO:0007275,GO:0040008	cytoplasm| auxin-activated signaling pathway| multicellular organism development| regulation of growth	ko04075	Plant hormone signal transduction
LOC100258858	IAMT1	indole-3-acetate O-methyltransferase 1	Down	GO:0042802,GO:0051749, GO:0000287,GO:0010252, GO:0009944	identical protein binding| indole acetic acid carboxyl methyltransferase activity| magnesium ion binding| auxin homeostasis| polarity specification of adaxial/abaxial axis		
HT5	STP6	hexose transporter	Down	GO:0005887,GO:0005886, GO:0009506,GO:0009679, GO:0005358,GO:0015145, GO:0046323,GO:0015749, GO:0009737,GO:0009651, GO:0009414	integral component of plasma membrane| plasma membrane| plasmodesma| hexose:proton symporter activity| high-affinity hydrogen:glucose symporter activity| monosaccharide transmembrane transporter activity| glucose import| monosaccharide transport| response to abscisic acid| response to salt stress| response to water deprivation		
LOC100242026	SAG12	senescence-specific cysteine protease SAG39	Down	GO:0010282,GO:0008234, GO:0007568,GO:0010150, GO:0010623,GO:0009737, GO:0009723,GO:0009739	senescence-associated vacuole| cysteine-type peptidase activity| aging| leaf senescence| programmed cell death involved in cell development| response to abscisic acid| response to ethylene| response to gibberellin		
LOC100246411	SAG12	senescence-specific cysteine protease SAG39	Down	GO:0010282,GO:0008234, GO:0007568,GO:0010150, GO:0010623,GO:0009737, GO:0009723,GO:0009739	senescence-associated vacuole| cysteine-type peptidase activity| aging| leaf senescence| programmed cell death involved in cell development| response to abscisic acid| response to ethylene| response to gibberellin		
LOC100247167	SAG12	senescence-specific cysteine protease SAG39	Down	GO:0010282,GO:0008234, GO:0007568,GO:0010150, GO:0010623,GO:0009737, GO:0009723,GO:0009739	senescence-associated vacuole| cysteine-type peptidase activity| aging| leaf senescence| programmed cell death involved in cell development| response to abscisic acid| response to ethylene| response to gibberellin		
LOC100257415	SAG12	senescence-specific cysteine protease SAG39	Down	GO:0010282,GO:0008234, GO:0007568,GO:0010150, GO:0010623,GO:0009737, GO:0009723,GO:0009739	senescence-associated vacuole| cysteine-type peptidase activity| aging| leaf senescence| programmed cell death involved in cell development| response to abscisic acid| response to ethylene| response to gibberellin		
LOC100252285	SAG12	senescence-specific cysteine protease SAG39	Down	GO:0010282,GO:0008234, GO:0007568,GO:0010150, GO:0010623,GO:0009737, GO:0009723,GO:0009739	senescence-associated vacuole| cysteine-type peptidase activity| aging| leaf senescence| programmed cell death involved in cell development| response to abscisic acid| response to ethylene| response to gibberellin		
LOC100264311	SAG12	senescence-specific cysteine protease SAG39	Down	GO:0005615,GO:0005764, GO:0010282,GO:0004197, GO:0007568,GO:0010150, GO:0010623,GO:0051603, GO:0009737,GO:0009723, GO:0009739	extracellular space| lysosome| senescence-associated vacuole| cysteine-type endopeptidase activity| aging| leaf senescence| programmed cell death involved in cell development| proteolysis involved in cellular protein catabolic process| response to abscisic acid| response to ethylene| response to gibberellin		
LOC100256178	LYK3	lysM domain receptor-like kinase 3	Down	GO:0016021,GO:0005886, GO:0005524,GO:0004675, GO:0009738,GO:0007166, GO:0006952,GO:0050832, GO:0045087,GO:0031348, GO:0009789,GO:0006468, GO:0009737	integral component of membrane| plasma membrane| ATP binding| transmembrane receptor protein serine/threonine kinase activity| abscisic acid-activated signaling pathway| cell surface receptor signaling pathway| defense response| defense response to fungus| innate immune response| negative regulation of defense response| positive regulation of abscisic acid-activated signaling pathway| protein phosphorylation| response to abscisic acid		
LOC100259233	NAC083	NAC domain-containing protein 83	Down	GO:0005634,GO:0003677, GO:0003700,GO:0010150, GO:0045892,GO:0009737, GO:0009651,GO:0006351, GO:0016032,GO:0010089	nucleus| DNA binding| transcription factor activity, sequence-specific DNA binding| leaf senescence| negative regulation of transcription, DNA-templated| response to abscisic acid| response to salt stress| transcription, DNA-templated| viral process| xylem development		
LOC100263051	NPF8.1	protein NRT1/PTR FAMILY 5.2	Down	GO:0016021,GO:0042936, GO:0042937,GO:0042742, GO:0042938,GO:0042538, GO:0009737,GO:0080052, GO:0009753,GO:0043201, GO:0080053,GO:0009751, GO:0009611,GO:0042939	integral component of membrane| dipeptide transporter activity| tripeptide transporter activity| defense response to bacterium| dipeptide transport| hyperosmotic salinity response| response to abscisic acid| response to histidine| response to jasmonic acid| response to leucine| response to phenylalanine| response to salicylic acid| response to wounding| tripeptide transport		
LOC100266572	GRDP1	glycine-rich domain-containing protein 2,	Down	GO:0005886,GO:0005198, GO:0009738,GO:0071470, GO:0009787,GO:0006979, GO:0009650	plasma membrane| structural molecule activity| abscisic acid-activated signaling pathway| cellular response to osmotic stress| regulation of abscisic acid-activated signaling pathway| response to oxidative stress| UV protection		
LOC100266913	ERF113	ethylene-responsive transcription factor ERF113	Down	GO:0005634,GO:0043565, GO:0003700,GO:0071497, GO:0009873,GO:0019760, GO:0045893,GO:0009737, GO:0009723,GO:0009753, GO:0009751,GO:0009651, GO:0009414,GO:0006351	nucleus| sequence-specific DNA binding| transcription factor activity, sequence-specific DNA binding| cellular response to freezing| ethylene-activated signaling pathway| glucosinolate metabolic process| positive regulation of transcription, DNA-templated| response to abscisic acid| response to ethylene| response to jasmonic acid| response to salicylic acid| response to salt stress| response to water deprivation| transcription, DNA-templated		
LOC104881446	SUGTL2	sugar transporter ERD6-like 3,	Up	GO:0005887,GO:0009705, GO:0005355,GO:0015145, GO:0005351,GO:0046323, GO:0035428,GO:0015749, GO:0009737,GO:0009651, GO:0009414	integral component of plasma membrane| plant-type vacuole membrane| glucose transmembrane transporter activity| monosaccharide transmembrane transporter activity| sugar:proton symporter activity| glucose import| hexose transmembrane transport| monosaccharide transport| response to abscisic acid| response to salt stress| response to water deprivation		
MFT	MFT	MFT-like protein	Up	GO:0005737,GO:0005634, GO:0009738,GO:0010030, GO:0009737	cytoplasm| nucleus| abscisic acid-activated signaling pathway| positive regulation of seed germination| response to abscisic acid		
LOC100232927	LHCA3	photosystem I chlorophyll a/b-binding protein 3-1, chloroplastic	Up	GO:0009507,GO:0009941, GO:0009534,GO:0009535, GO:0016021,GO:0016020, GO:0009522,GO:0010287, GO:0009579,GO:0016168, GO:0046872,GO:0031409, GO:0009768,GO:0018298, GO:0009409,GO:0009735, GO:0009644,GO:0009645	chloroplast| chloroplast envelope| chloroplast thylakoid| chloroplast thylakoid membrane| integral component of membrane| membrane| photosystem I| plastoglobule| thylakoid| chlorophyll binding| metal ion binding| pigment binding| photosynthesis, light harvesting in photosystem I| protein-chromophore linkage| response to cold| response to cytokinin| response to high light intensity| response to low light intensity stimulus	ko00196	Photosynthesis−antenna proteins
LOC100257475	LOG4	probable cytokinin riboside 5′-monophosphate phosphoribohydrolase LOGL10,	Down	GO:0005737,GO:0005634, GO:0070694,GO:0043733, GO:0016799,GO:0070635, GO:0070636,GO:0000701, GO:0017065,GO:0009691	cytoplasm| nucleus| deoxyribonucleoside 5′-monophosphate N-glycosidase activity| DNA-3-methylbase glycosylase activity| hydrolase activity, hydrolyzing N-glycosyl compounds| nicotinamide riboside hydrolase activity| nicotinic acid riboside hydrolase activity| purine-specific mismatch base pair DNA N-glycosylase activity| single-strand selective uracil DNA N-glycosylase activity| cytokinin biosynthetic process		
LOC100232996	GA20OX2	gibberellin 20-oxidase	Down	GO:0005737,GO:0045544, GO:0046872,GO:0009908, GO:0009740,GO:0009686, GO:0048366,GO:0009739, GO:0048575,GO:0009826	cytoplasm| gibberellin 20-oxidase activity| metal ion binding| flower development| gibberellic acid mediated signaling pathway| gibberellin biosynthetic process| leaf development| response to gibberellin| short-day photoperiodism, flowering| unidimensional cell growth	ko00904	Diterpenoid biosynthesis
LOC100257500	GA20OX2	gibberellin 20 oxidase 1-D-like	Up	GO:0045544,GO:0046872, GO:0009908,GO:0009740, GO:0009686,GO:0009739, GO:0080167,GO:0009639, GO:0009826	gibberellin 20-oxidase activity| metal ion binding| flower development| gibberellic acid mediated signaling pathway| gibberellin biosynthetic process| response to gibberellin| response to karrikin| response to red or far red light| unidimensional cell growth	ko00904	Diterpenoid biosynthesis
LOC100854724	GA20OX3	gibberellin 20 oxidase 1	Down	GO:0005737,GO:0045544, GO:0046872,GO:0009908, GO:0009740,GO:0009686, GO:0048366,GO:0009739, GO:0048575,GO:0009826	cytoplasm| gibberellin 20-oxidase activity| metal ion binding| flower development| gibberellic acid mediated signaling pathway| gibberellin biosynthetic process| leaf development| response to gibberellin| short-day photoperiodism, flowering| unidimensional cell growth		
LOC100259502	MYB21	transcription factor MYB24	Down	GO:0005634,GO:0003677, GO:0003700,GO:0016036, GO:0009686,GO:0009751	nucleus| DNA binding| transcription factor activity, sequence-specific DNA binding| cellular response to phosphate starvation| gibberellin biosynthetic process| response to salicylic acid		
LOC100260318	MYB27	transcription factor MYB59	Down	GO:0005634,GO:0003677, GO:0003700,GO:0046686, GO:0010200,GO:0009723, GO:0009739,GO:0009753, GO:0009751,GO:0006351	nucleus| DNA binding| transcription factor activity, sequence-specific DNA binding| response to cadmium ion| response to chitin| response to ethylene| response to gibberellin| response to jasmonic acid| response to salicylic acid| transcription, DNA-templated		
LOC100263072	MYB48	protein ODORANT1	Down	GO:0005634,GO:0003677, GO:0003700,GO:0046686, GO:0010200,GO:0009723, GO:0009739,GO:0009753, GO:0009751,GO:0006351	nucleus| DNA binding| transcription factor activity, sequence-specific DNA binding| response to cadmium ion| response to chitin| response to ethylene| response to gibberellin| response to jasmonic acid| response to salicylic acid| transcription, DNA-templated		
LOC100241060	BBM2	AP2-like ethylene-responsive transcription factor BBM	Down	GO:0005634,GO:0003677, GO:0003700,GO:0007275, GO:0006351	nucleus| DNA binding| transcription factor activity, sequence-specific DNA binding| multicellular organism development| transcription, DNA-templated		
LOC100245800	ERF011	ethylene-responsive transcription factor ERF016	Up	GO:0005634,GO:0003677, GO:0003700,GO:0009873, GO:0006351	nucleus| DNA binding| transcription factor activity, sequence-specific DNA binding| ethylene-activated signaling pathway| transcription, DNA-templated		
LOC104879942	ERF109	ethylene-responsive transcription factor ERF109-like	Up	GO:0005634,GO:0003677, GO:0003700,GO:0009873, GO:0006351	nucleus| DNA binding| transcription factor activity, sequence-specific DNA binding| ethylene-activated signaling pathway| transcription, DNA-templated		
LOC100250476	ERF113	ethylene-responsive transcription factor ERF113	Down	GO:0005634,GO:0043565, GO:0003700,GO:0050832, GO:0009873,GO:0007275, GO:0006351	nucleus| sequence-specific DNA binding| transcription factor activity, sequence-specific DNA binding| defense response to fungus| ethylene-activated signaling pathway| multicellular organism development| transcription, DNA-templated		
LOC100259623	ERF5	ethylene-responsive transcription factor ERF104-like	Down	GO:0005634,GO:0003677, GO:0003700,GO:0009873, GO:0010200,GO:0006351	nucleus| DNA binding| transcription factor activity, sequence-specific DNA binding| ethylene-activated signaling pathway| response to chitin| transcription, DNA-templated		
LOC100251140	ERF095	ethylene-responsive transcription factor ERF095	Down	GO:0005634,GO:0003677, GO:0003700,GO:0009873, GO:0006351	nucleus| DNA binding| transcription factor activity, sequence-specific DNA binding| ethylene-activated signaling pathway| transcription, DNA-templated	ko04016,ko04075	MAPK signaling pathway−plant| Plant hormone signal transduction
LOC100255447	ERF062	ethylene-responsive transcription factor ERF062-like,	Down	GO:0005634,GO:0003677, GO:0003700,GO:0009873, GO:0006351	nucleus| DNA binding| transcription factor activity, sequence-specific DNA binding| ethylene-activated signaling pathway| transcription, DNA-templated		
LOC100852555		2-methylene-furan-3-one reductase	Up	GO:0048046,GO:0009941, GO:0009570,GO:0010319, GO:0009579,GO:0035798, GO:0035671,GO:0008270, GO:0009409	apoplast| chloroplast envelope| chloroplast stroma| stromule| thylakoid| 2-alkenal reductase (NADP+) activity| enone reductase activity| zinc ion binding| response to cold		

**FIGURE 5:**
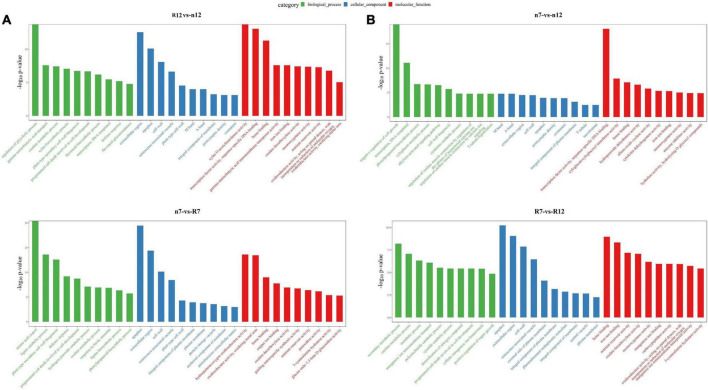
Gene ontology (GO) enrichment analysis results between different comparison groups **(A)** R12 vs. n12, n7 vs. R7. **(B)** n7 vs. n12, R7 vs. R12. n7, R7, n12, and R12 represent 70 DAP and 125 DAP sampling time points under nR and RR cultivation.

In omics research, following the acquisition of transcriptome data, GO enrichment analysis is mainly utilized to address the issue of investigating a large number of molecular changes. GO enrichment analysis can categorize thousands of molecules (such as proteins or specific non-coding RNAs) based on their functional similarity and then evaluate them, significantly lowering the cost and difficulty of analysis. Therefore, GO enrichment analysis was also performed on the transcriptome data obtained in this study, and the results are shown in Figure 5. The GO enrichment analysis of the DEGs between different the comparison groups indicated that they were involved in various biological processes, cellular components, and molecular functions. In terms of nR7 vs. RR7, we discovered that amino acid export, apoplast, and hydroquinone:oxygen oxidoreductase activity were critical biological processes, cellular components, and molecular activities, respectively. For nR12 vs. RR12, the regulation of the glycolytic process, the extracellular region, and xylan O-acetyltransferase activity were the main biological processes, cellular components, and molecular functions. The top three cell components were the apoplast, extracellular region, and cell wall. Hydroquinone:oxygen oxidoreductase activity and oxidizing metal ions were the top molecular functions in nR7 vs. RR7, whereas xylan O-acetyltransferase activity and transcription factor activity were the top molecular functions in nR12 vs. RR12 (Figure 5A).

Further exploration of the effects of different growth stages on biological processes, cellular components, and molecular functions indicated that the natural processes of nR7 vs. nR12 were mainly enriched in the negative regulation of cell growth and transcription of DNA as a template. Moreover, the ethylene-activated signaling pathway and cytokinin catabolic process were implicated. The most enriched cell component was the M band, and the most enriched molecular function was transcription factor activity. In addition, compared with those of the other comparison groups, the biological processes of the RR7 vs. RR12 group had higher enrichment in secondary metabolic processes, oxalate metabolic processes, and metabolic processes. The most enriched cellular component was the apoplast, and the most enriched molecular function was heme binding (Figure 5B).

### Combined transcriptome and microRNA sequencing analysis

**FIGURE 6:**
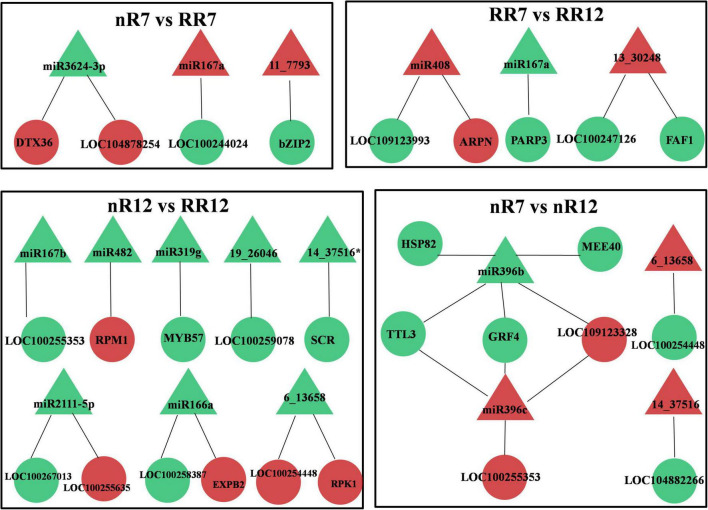
Conjoint analysis of differentially expressed miRNAs and differentially expressed target genes in different groups, including nR7 vs. nR12, RR7 vs. RR12, nR7 vs. RR7, and nR12 vs. RR12. Red indicates up-regulated expression, while green indicates down-regulated expression. nR7, RR7, nR12, and RR12 represent the 70 DAP and 125 DAP sampling time points under nR and RR cultivation. Circles represented DEGs and triangles represented DEMs.

Since the transcriptome data and previously published miRNA sequencing were collected from the same root samples of grapevine cv. “Muscat Hamburg,” a combined analysis of the differentially expressed miRNAs (DEMs) and DEGs was conducted. The results demonstrated that in each comparison group (nR7 vs. RR7, nR12 vs. RR12, nR7 vs. nR12, and RR7 vs. RR12), both DEMs and predicted targeted DEGs were found. Each DEM had one or two target DEGs, and the number of DEGs related to the DEMs was mainly 1-2, albeit with 5 and 4 differentially expressed target genes of *miR396b* and *miR396c*, respectively (Figure 6).

*miR3624-3p*, *miR167a/b*, *miR482*, *miR319g*, *miR2111-5p*, and *miR166a* were found to be DEMs of the DEGs obtained from the roots of plants in the various comparison groups (nR7 vs. RR7 and nR12 vs. RR12). The target gene of *miR3624-3p* was the protein DETOXIFICATION 36 (*DTX36*, *LOC100265485*), and its product was a detoxified protein (mulatexin, *LOC104878254*). RPM1 is a disease resistance protein targeted by *miR482*, and the target gene of *miR319g* is the transcription factor MYB57 (MYB57, *LOC104878254*). In addition, there were novel miRNAs, namely, *11_7793*, *19_26046*, and 14_37516*, that were differentially expressed. Among these, the target gene of *11_7793* encoded the transcription factor bZIP2 (bZIP2, *LOC104878442*), and the target gene of *6_13658* encoded the LRR receptor-like serine/threonine-protein kinase. The DEMs and predicted targeted DEGs were also analyzed at different developmental stages (nR7 vs. nR12 and RR7 vs. RR12). The known DEMs were *miR167a*, *miR408*, and *miR396b/c*. The basic blue protein mavicyanin-like (ARPN, *LOC109124259*), which acts in redox processes, was *miR408*’s target gene. *MiR396b/c* targets include growth-regulating factor 3 (GRF4, *LOC100242152*) and heat shock cognate protein 80 (HSP82, *LOC100246843*). Moreover, the novel DEMs were *6_13658*, *14_37516*, and *13_30248*, among which the target gene protein FANTASTIC FOUR 3 (*FAF1*, *LOC100252736*) of 13_30248 functions in the regulation of meristematic activity (Figure 6).

Fragments per kilobase per million reads is typically used to represent gene expression, and the calculation method of TPM is very similar to that of RPKM. The total TPM of each sample was the same after the gene length and sequencing depth were normalized (both were 10). This means that the TPM number reflects the proportion of reads compared to a gene, allowing direct comparison between samples. This fact also suggests that, compared with the FPKM method, the standardized method of TPM is more advantageous, and its standardization is currently recommended. Further analysis of the TPM and FPKM values of the known miRNAs and their target DEGs under root restriction cultivation in the four sequencing samples revealed that the expression trends of the DEMs and DEGs were opposite in the nR7 vs. RR7 and nR12 vs. RR12 comparison groups. For example, the expression of *miR3624-3p* in RR7 and RR12 increased after root restriction cultivation compared with that of nR7 and nR12 in the same period. The target gene protein DETOXIFICATION 41 (*DTX36*, *LOC100255800*) showed downregulated expression, and mulatexin (MYB57 transcription factor, LOC104878254) showed upregulated expression. For *miR167b*, we found that the target gene putative germin-like protein 9-2 (*LOC100255353*) showed upregulated expression in RR12 and downregulated expression in nR7, RR7, and nR12. For *miR167a*, we found that the target gene ankyrin repeat-containing protein (*LOC100244024*) showed upregulated expression in RR7, nR12, and RR12.

**FIGURE 7:**
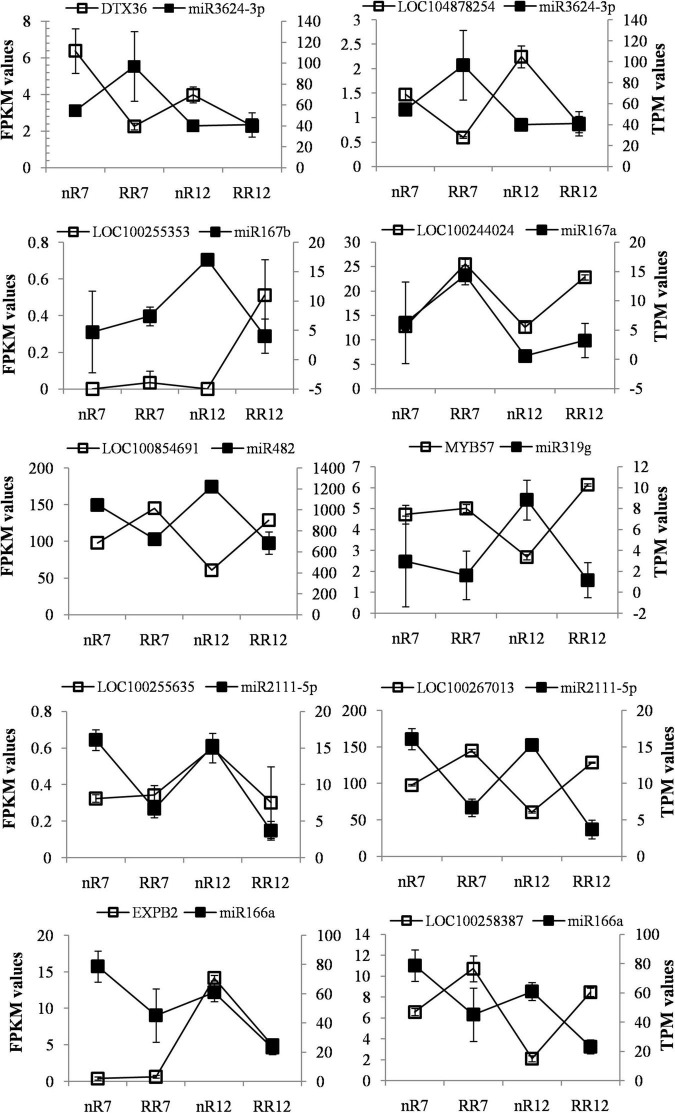
FPKM and TPM values of differential expression known miRNAs and their target genes under different cultivation methods comparing groups of the nR7 vs. RR7 and nR12 vs. RR12 in four sequencing samples. nR7, RR7, nR12, and RR12 represent the 70 days DAP and 125 DAP sampling points under nR and RR cultivation.

Moreover, the expression of *miR482* in nR7 and nR12 was upregulated, and its target gene disease resistance protein RPM1 (*LOC100854691*) showed an upregulated trend in RR7 and RR12. In nR12, *miR319g* expression was increased while its target gene, mulatexin (MYB57 transcription factor, *LOC104878254*), was more highly expressed in nR7, RR7, and RR12. Furthermore, the results also indicated that *miR2111-5p* had two target genes (*LOC100255635*, *LOC100267013*), and their expression was upregulated in nR7 and nR12. *MiR166a* expression was similarly upregulated in nR7, and its target gene *putative expansin-B2* (*EXPB2*, *LOC100253046*) was upregulated in nR12; however, the target gene *glucan endo-1,3-beta-glucosidase 11* (*Glc5-1-11*, *LOC100258387*) was exclusively upregulated in RR7 and RR12 (Figure 7).

### Effect of root restriction treatment on the endogenous phytohormone contents of grapevine roots

**FIGURE 8:**
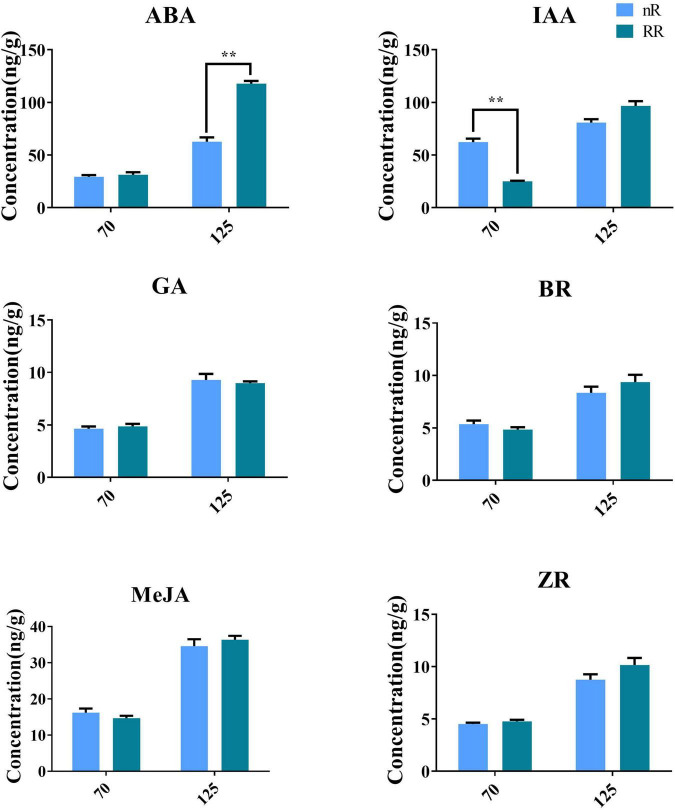
Effect of RR treatment and nR treatment on the contents of endogenous phytohormones in grapevine roots of different developmental stages. A total of six phytohormones were detected in this study, including ABA, IAA, MeJA, GA, BR, and ZR. The ** indicated whether there is a significant difference between the two treatments.

The endogenous contents of the phytohormones measured in the grapevine roots of the RR group and the nR group are shown in Figure 8. RR treatment significantly increased the ABA content at 125 DAA and significantly inhibited IAA accumulation in the roots at 70 DAA, but RR treatment had no significant effect on the contents of MeJA, GAs, ZR, or BR.

### qRT-PCR-based validation of differentially expressed genes involved in the abscisic acid biosynthesis and metabolic pathways

**FIGURE 9:**
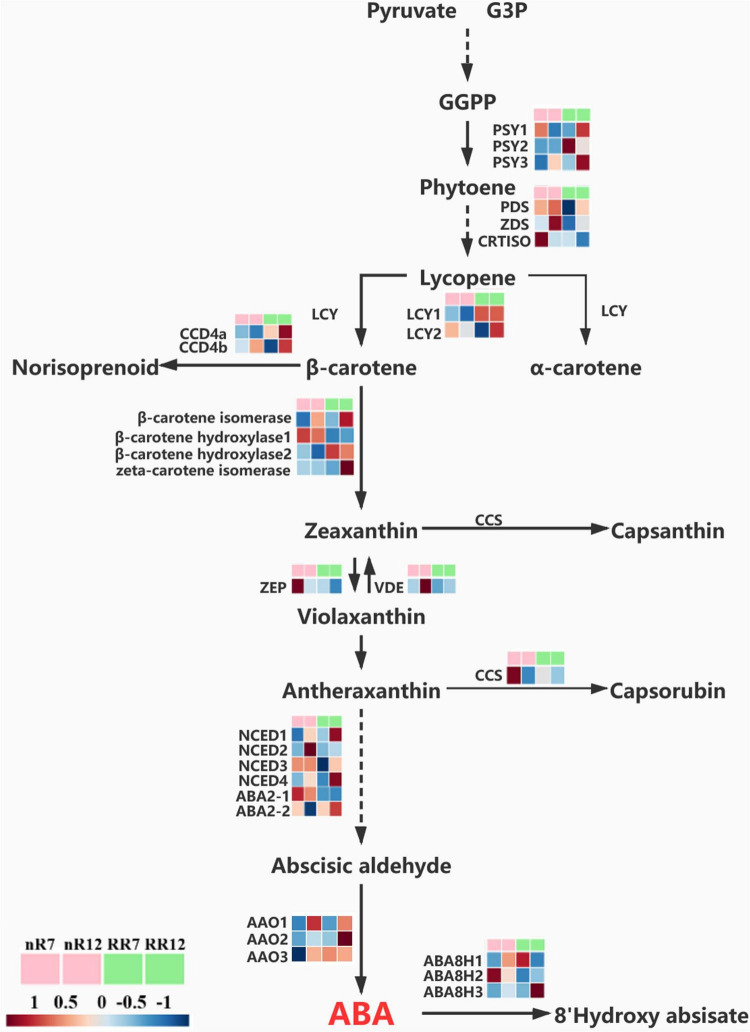
Relative expression levels of genes in ABA biosynthesis and metabolism pathway. The expression levels of a total of 26 genes under different cultivation modes (nR and RR) and at different developmental stages (70 DAP and 125 DAP) are shown in heat maps, with red representing up-regulation and blue representing down-regulation.

Because the ABA content under RR cultivation was significantly higher than that in the control group at the late stage of root development, the expression pattern of genes involved in ABA biosynthesis was also quantified further to understand the effects of RR cultivation on ABA biosynthesis at the transcriptional level. Twenty-six DEGs involved in the ABA pathway were identified and visualized within a path map (Figure 9). The genes *VvPSY1* and *VvPSY3* (responsible for GGPP to phytoene conversion) were upregulated in RR12, as were the genes *LCY1* (responsible for carotene biosynthesis) in both RR7 and RR12 and the genes *CCD4a* and *CCD4b* (responsible for norisoprenoid biosynthesis) in RR12. In addition, genes contributing to zeaxanthin formation (β*-carotene isomerase*, β*-carotene hydroxylase 2*, *zeta-carotene isomerase*) were expressed at a higher level in RR12 than in the other groups. In comparison, genes contributing to zeaxanthin metabolism (*ZEP*) were expressed at a higher level in nR7 than in the other groups. Interestingly, most genes directly related to ABA biosynthesis (*NCED1*, *NCED3*, *NCED4*, *ABA2-2*) were upregulated in RR12, while genes related to ABA metabolism (*ABA8H1*, *ABA8H2*) were upregulated in nR12.

### qRT-PCR-based validation of differentially expressed genes involved in the auxin biosynthesis and metabolic pathways

**FIGURE 10:**
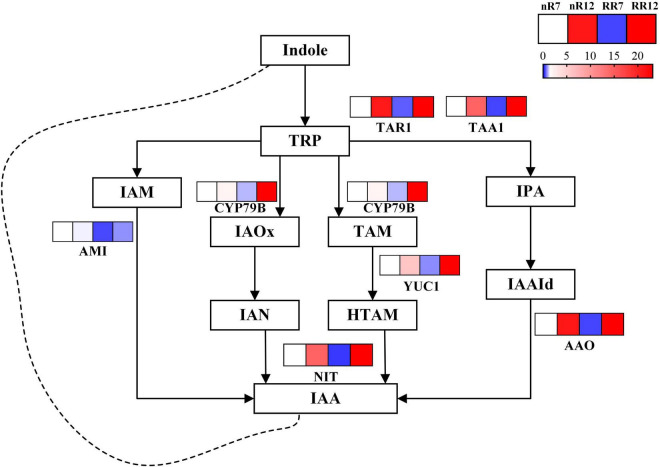
Relative expression levels of genes in IAA biosynthesis and metabolism pathway. The expression levels of a total of 7 genes under different cultivation modes (nR and RR) and at different developmental stages (70 DAP and 125 DAP) are shown in heat maps, with red representing up-regulation and blue representing down-regulation.

Twenty-six DEGs involved in the IAA pathway were identified and visualized within a path map (Figure 10). The genes *TAR1* and *TAA1* (responsible for the conversion of indoles to IPA), *CYP79B* (responsible for TRP catabolism), *YUC1* (responsible for TAM catabolism), *AAO* (responsible for IAAId to IAA conversion) and *NIT* (responsible for IAN/HTAM to IAA conversion) were downregulated in RR7 and upregulated in RR12. In contrast, the gene *AMI* (responsible for IAM to IAA conversion) was downregulated in RR7, nR12 and RR12, while the decreasing trend was more obvious in nR7 than in the other groups.

## Discussion

### Transcriptome sequencing revealed the expression profiles of root phytohormones-related genes under different cultivation modes

The sequencing depth and repeatability were indexed to evaluate the sequencing quality. For the transcriptome sequencing results obtained in our study, the amount of high-quality data of each sequenced sample was greater than 5 GB worth, the Q30 base distribution was greater than 92%, and the rate of obtained clean reads that mapped to the grapevine genome was greater than 90%. Taken together, all of these results illustrated that the quality and depth of the samples were high. Furthermore, the correlation coefficient between the replicates of each root sample was more than 0.99, reflecting the excellent repeatability between the samples.

In this study, 2799 DEGs were discovered in the nR12 vs. RR12 comparison group, the number of which was much greater than the 1,532 DEGs discovered in the nR7 vs. RR7 comparison group. These results indicated that other variations occurred at the transcriptome level, aside from the more visible root phenotypic changes associated with the root length following RR culture. This was also consistent with the finding of more DEMs in the root samples 125 days after planting under RR cultivation following miRNA sequencing. There were more downregulated genes than upregulated genes, which was more evident in the different nR7 vs. nR12 and RR7 vs. RR12 developmental stage comparison groups.

Gene Ontology enrichment analysis of the DEGs revealed that multiple genes respond to the process of phytohormone signal transduction. As an essential endogenous regulator of plant growth, phytohormones are active substances that can regulate plant physiological responses at low concentrations and are synthesized in plant cells receiving specific environmental signals. Among them, IAA, ABA, CTK, GAs, and ETH play essential roles in root development in some or all stages of plant growth. Specifically, IAA participates in all aspects of plant root growth, including regulating the growth of the primary roots, lateral roots, adventitious roots, root hairs, and vascular tissue (Bhalerao et al., 2002). Several DEGs (i.e., PINs) can affect the IAA distribution in the roots, play an essential role in the transport of intercellular IAA, and regulate gravitropism (Kleine-Vehn et al., 2010). ABA is another important phytohormone related to plant stress that mediates the plant responses to environmental factors, such as temperature, nitrate, and water stress (Lee and Luan, 2012; Hong et al., 2013). These environmental factors can affect plant growth, especially that of the root structure, and promote the growth of roots in one area while inhibiting the growth of roots in another area (Zhu et al., 2010). Many studies have reported that ABA contributes greatly to root development in model plant species, and ectopic expression of *OBP4* or loss of *RSL2* function in *Arabidopsis thaliana* has been shown to lead to ABA-insensitive root hair growth, confirming that OBP4-mediated RSL2 transcriptional inhibition contributes to ABA-dependent inhibition of root hair growth (Rymen et al., 2017). In this study, qRT-PCR showed that RR induced the expression of most genes involved in the ABA biosynthesis pathway, which might also be a factor in configuration changes, such as the increase in the number of fine roots. In addition, the crosstalk between several endogenous phytohormones also influences the changes in root architecture; IAA might affect ETH accumulation in root biomass independently, and the sensitivity of *Arabidopsis thaliana* to ETH also increases the reactivity of IAA (Alla et al., 2018). The *Arabidopsis thaliana* dysfunctional mutant *ACC oxidase 2* and *constitutive triplet reaction 1* (ETH signaling) is defective in terms of the ABA-induced reversal of the inhibition of seed germination mediated by 1*-aminocyclopropane-1-carboxylic acid* (ACC). In contrast, ETH production by the ACC oxidase orthologs Lepidium ACO2 and Arabidopsis ACO2 appears to be a critical regulatory step (Linkies et al., 2009). One phytohormone often stimulates the biosynthesis of other phytohormones or alters their distribution (Müller, 2021). In this study, several genes identified in this work responded to the activity of various phytohormone pathways, namely, PIN2 and PIN6 in response to IAA and ETH simultaneously, ERF113 in response to ETH and ABA, and MYB27 and MYB48 transcription factors in response to GAs and ETH. Most of the downstream genes of IAA biosynthesis were upregulated in RR7 and downregulated in RR12 and were associated with the ratio of IAA concentration under RR treatment to the control increasing from 2:1 to 1:1.2, which may be one of the factors responsible for RR-induced changes in root architecture (increased number and ratio of fine roots). The above results provide reference information for verifying the interaction of multiple phytohormones in regulating grape root development, and the DEGs identified in this study are worthy of further research on the function of phytohormone interactions during root development.

### Several microRNAs and their target genes played an antagonistic role in the regulation of grape root development

MicroRNAs (miRNAs) play essential roles in plant growth, development, and response to stress, and numerous studies in different plant species have confirmed that miRNAs regulate gene expression and traits by regulating target genes, thus controlling the variation in plant phenotypes (Gao et al., 2019). Plant roots absorb water and nutrients and therefore are the sites through which plants interact with the soil environment. Some miRNAs and their targets, such as miR164 and its plant-specific transcription factor, are involved in plant lateral root growth. NAC1 DOMAIN-CONTAINING PROTEIN 1 (NAC1), which regulates lateral root formation in Arabidopsis and maize, has been identified (Jiang et al., 2019; Singh et al., 2021). In this study, GO analysis of the transcriptome sequencing data and miRNA sequencing data found that miRNAs AR important in root development; specifically, *MiR165/166* can be used as a positional cue for regulating root tip meristem. Previous studies in Arabidopsis have found that, after being synthesized in the vascular column, the short root (SHR) protein can activate the transcription of *miR165A* and *miR166B*, while *miR165/166* regulates root apical meristems. SHR also affects primary root growth through non-cell-autonomous restriction of HD-ZIP III transcription factors (Carlsbecker et al., 2010). In addition, *miR396* was found to target the expression of growth regulatory factors and affects the size of root meristems (Liu et al., 2012). Moreover, miR408 can target the *CUPREDOXIN* gene to regulate root cap formation, lateral root development, and root elongation (Walch-Liu et al., 2006). The auxin-responsive factors ARF6 and ARF8 are positive regulators that promote the growth of plant adventitious roots, and miR167 strictly regulates both. In contrast, the targets of *miR160* and ARF17 (auxin-responsive factor) are negative regulators that control adventitious rooting. These three ARF proteins have overlapping expression domains that interact with genes and regulate each other’s expression at the transcriptional and posttranscriptional levels through regulation of the availability of *miR160* and *miR167* (Gutierrez et al., 2009). Previous studies have also demonstrated that *miR2111* is the only miRNA somewhat expressed in stems, and its presence in roots is mainly due to long-distance transport and affects the occurrence of root nodules in legumes. Furthermore, overexpression of *miR2111* in Arabidopsis can promote the development of primary roots and lateral roots (Tsikou et al., 2018). Taken together, these findings have laid a solid foundation for further research on the regulatory network of miRNAs regulating grapevine root development through target genes. In this study, by combining the transcriptome sequencing results and miRNA sequencing results obtained earlier in our laboratory, we concluded that a large number of miRNAs and their target genes play an antagonistic role in the regulation of grape root development.

### Root-restriction cultivation indeed hugely influenced the formation of abscisic acid and auxin in grapevine roots

Root-restriction treatment has been reported to affect the formation of endogenous phytohormones in different grape organs and plays a crucial role in promoting ABA accumulation as well as inhibiting IAA biosynthesis (Li et al., 2022c). To further verify the changes in endogenous phytohormone contents in the roots at different developmental stages under the two cultivation treatments, the contents of six plant hormones, including ABA and IAA, were measured in this study. The results strongly supported those of previous studies, confirming that there were significant differences in the contents of ABA and IAA under the two different cultivation treatments, while the contents of the other four phytohormones exhibited no significant differences. Previously, through quantitative expression analysis of ABA-related rate-limiting enzyme genes in different tissues, we discovered that that *VvPYL1* exhibited the highest expression in the roots. Spatiotemporal expression analysis showed that *VvPYL1* was highly expressed in stages II and III and that *VvPYL1* was highly expressed in the lateral roots of grape seedlings. Overexpression of *VvPYL1* in *Arabidopsis thaliana* resulted in root hairs longer than those of the wild type, and the root hair length of transgenic lines was affected by exogenous ABA (Li et al., 2020b). In addition, qRT-PCR analysis of the key genes of the IAA biosynthesis pathway, including *VvTAR1*, *VvTAA1*, *VvCYP79B*, *VvYUC1*, *VvAAO* and *VvNIT*, revealed that most were significantly downregulated under RR cultivation at the early stage of root development (RR7). Previously, our expression analysis of IAA-related rate-limiting enzyme genes in different organs revealed that *VvNIT* as well as *VvAAO*, which directly determine IAA biosynthesis, had the highest expression in the roots, and RR treatment could significantly inhibit its accumulation at the early development stage of the roots, leading to a significant difference in grapevine phenotypes (Li et al., 2022c). Overall, the above findings suggested that RR cultivation could regulate the expression of the root hair elongation-related gene *VvPYL* by increasing endogenous ABA contents while regulating the expression of the IAA biosynthesis-related genes *VvNIT* and *VvAAO* to regulate steady-state IAA levels, giving rise to root architecture changes of grape seedlings as well as growth differences in grapevine. Because IAA can promote rooting, the effect of ABA on rooting is not clear (Zhang et al., 2022). By combining the data concerning the contents of ABA and IAA obtained and the qRT–PCR data in this study, we also made a reasonable assumption that ABA and IAA had a synergistic effect in the process of mediating grape rooting: IAA played a role in the early stage of root development, while ABA played a role in the late stage of root development. However, the specific underlying molecular mechanism remains unknown.

## Conclusion

Overall, the results indicated that there were 2031, 1445, 1532, and 2799 DEGs in the various comparison groups (nR7 vs. nR12, RR7 vs. RR12, nR7 vs. RR7, and nR12 vs. RR12, respectively). Compared with the root system at 70 DAP, the root system under RR cultivation at 125 DAP exhibited greater phenotypic differences and significantly increased numbers of DEGs. By combining the results of our analysis of the transcriptome data and previously published miRNA sequencing data, we found that DEM and DEG combinations in different comparison groups showed opposite trends, as which most DEGs were expressed at a higher level than DEMs. Further investigation indicated the accumulation of IAA was inhibited at the early stage of root development, while the formation of ABA was promoted at the later stage of root development, eventually caused the differences in root architecture under the two cultivation regimes. Moreover, different genes related to IAA and ABA biosynthesis were expressed at different levels under the different cultivation treatments. Taken together, these findings lay a foundation for further investigation of the DEGs responsible for root architecture changes under RR cultivation, providing theoretical reference information for future research in potential binding targets of miRNAs to elucidate the molecular mechanisms of transcriptional and posttranscriptional regulation on grape root development. Moreover, studies investigating the specific molecular mechanism through which endogenous ABA/IAA contents affect root architecture need to be further carried out.

## Data availability statement

The datasets presented in this study can be found in online repositories. The names of the repository/repositories and accession number(s) can be found in the article/Supplementary material.

## Author contributions

LW and HL designed the research. JL and GL implemented and related the qRT-PCR experiments. HL, JL, and XL analyzed the data, composed the manuscript, and drew the figures. JL and MZ reviewed and revised the manuscript. DL, CM, WX, SS, and SW reviewed the manuscript. XL and SW took responsibility for this experiment. All authors contributed to the article and approved the submitted version.

## Footnotes

1
https://www.ncbi.nlm.nih.gov/data-hub/taxonomy/3603/

2
http://plants.ensembl.org/info/about/index.html

3
https://biodb.swu.edu.cn/qprimerdb/browse_plants

## Supplementary material

Supplementary Figure 1 Grapevine roots pictures of different treatment groups (nR7, RR7, nR12, RR12).

Supplementary Table 1 Primers for qRT-PCR quantification. The sequences of genes in ABA and IAA biosynthesis pathways are all obtained in EnsemblPlants (http://plants.ensembl.org/info/about/index.html).
