# Supplementary figures and images for: Transcriptome analysis revealed the expression levels of genes related to abscisic acid and auxin biosynthesis in grapevine (Vitis vinifera L.) under root restriction
Source: Front Plant Sci. 2022 Aug 24;13:959693. doi: 10.3389/fpls.2022.959693 (PMC9449541; doi:10.3389/fpls.2022.959693)

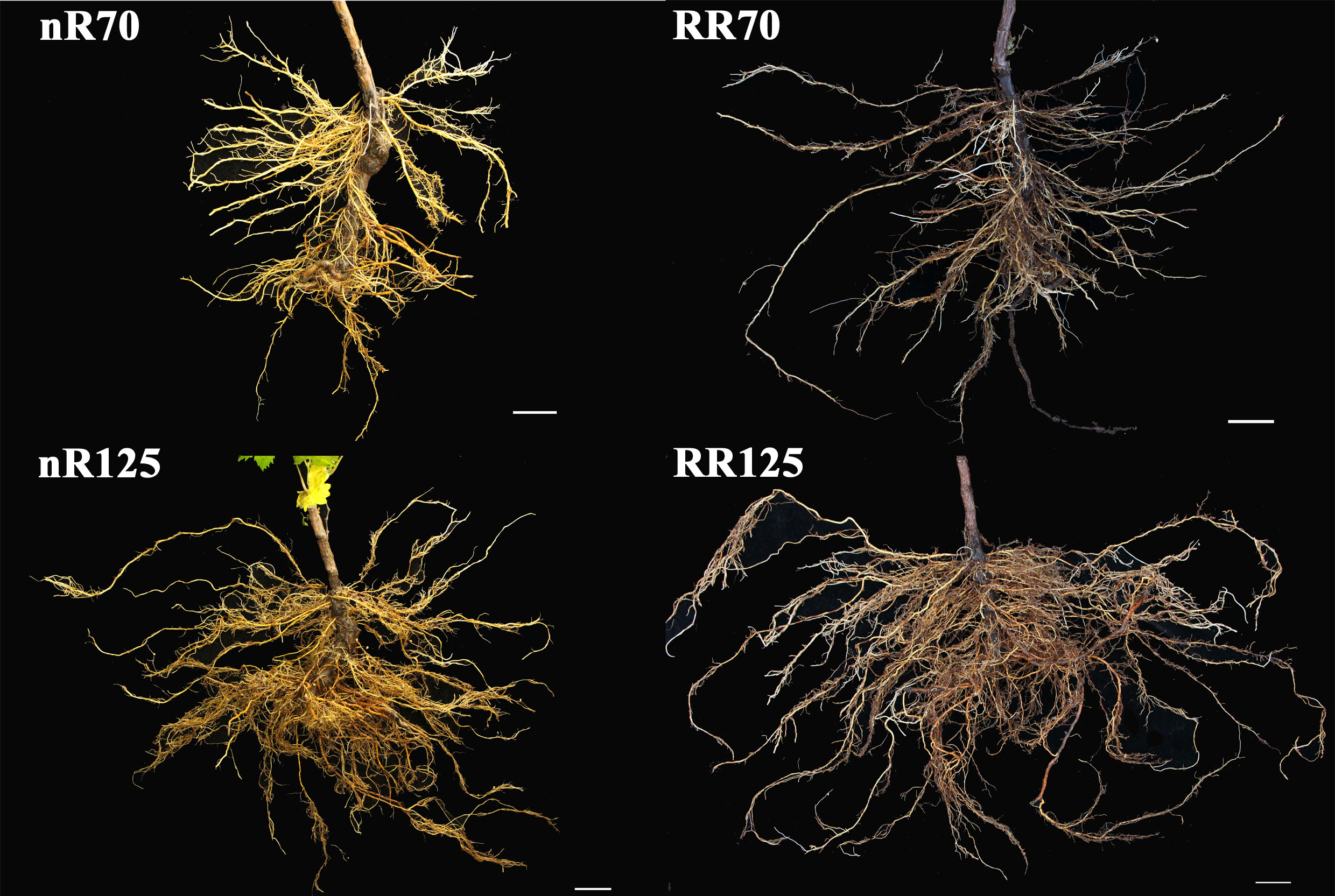

Supplement: Supplementary Figure 1 — Grapevine roots pictures of different treatment groups (nR7, RR7, nR12, RR12). [file Image_1.JPEG]
