# Supplementary material for: Transcriptome analysis revealed the expression levels of genes related to abscisic acid and auxin biosynthesis in grapevine (Vitis vinifera L.) under root restriction
Source: Front Plant Sci. 2022 Aug 24;13:959693. doi: 10.3389/fpls.2022.959693 (PMC9449541; doi:10.3389/fpls.2022.959693)
Supplement: Supplementary Table 1 — Primers for qRT-PCR quantification. The sequences of genes in ABA and IAA biosynthesis pathways are all obtained in EnsemblPlants (http://plants.ensembl.org/info/about/index.html). [file Table_1.docx]

**Table S1.** Primers for qRT-PCR quantification, the sequences of genes in ABA and IAA biosynthesis pathways were all obtained in EnsemblPlants (http://plants.ensembl.org/info/about/index.html).

| **Target genes** | **Forward sequence（5’ → 3’）** | **Reverse Sequence（5’ → 3’）** |
| --- | --- | --- |
| *Vvactin* | GACAATTTCCCGTTCAGCAGT | GATTCTGGTGATGGTGTGAGT |
| *VvPSY1* | GACAAGGCTTCCATGCTTGATGAGG | TCTCATCTGGGAAAGTTGGACAGGC |
| *VvPSY2* | ACACCAAGCCTTGCTCTTCTTCTCC | GGCATAAGTTTGGCATTGTGAAGCG |
| *VvPSY3* | AACAAGACAGACAAGGCTTCCATGC | GGCAGTACTCCAGGTAGACACATAGG |
| *VvPDS* | CAGAAACCCGAAGACATTGCAGAGG | TCGAGCTCAAGAAACTTGTTCACCC |
| *VvZDS* | AACCACACCGCTTCACAATTACACC | TATCCTCCTCGGATGTCCATGCTCC |
| *VvLCY1* | AGAAACCCGAAGACATTGCAGAGGG | ATCGAGCTCAAGAAACTTGTTCACCC |
| *VvLCY2* | GAGTTCAAGGCCGAAGCGAATTAGG | AAGATTGGGATATGACGGTCGAGGG |
| *VvCCD4A* | ATGCTGCACTTGCCTATGATGAAGC | AAGCTGTTGTTGCTGAGGAAGAAGG |
| *VvCCD4B* | AGGTTCAAAGGAAACAAAGCCAAGC | ATGGATGAGGAAGAGGAGGAACAGC |
| *VvZEP* | ATGGTAAAGGAGGCAGAGGAGTTCG | CTTGTCTGCAAGCTTGTCTCTGTCG |
| *VvVDE* | ACGCACCATCTTTGATGTTAAGCGG | TGGCTTCCCTTCTTTGTTCACAACC |
| *VvCCS* | AACGATCAAGGCAACAGAATCACCC | AGCCGCTTAACATCAAAGATGGTGC |
| *VvCRTISO* | CGGTGACCAATTTCGAGATGAACCG | CTGATTCAAGCGAGAGCTTTAGCCG |
| *Vvβ-carotene1* | CTCACATCTACGACCATGGTTCTGC | ACGATGAGTCTTCCACTTCCGATCC |
| *Vvβ-carotene2* | CTCAACTCCATCACCAACAGGTTCC | ATCTTGGGTCTCTGTTTGGCTCTCC |
| *Vvβ-carotene3* | TCTCACTTCAGATTCCACTGCTCCC | TTCATGCCCAAACCCTTCATGTTCC |
| *Vvβ-carotene4* | TTTGGTGTAAGCAAGAGCAAGACCC | GAGGTTGTGCAGAACATTAGGCTGG |
| *VvTAA1* | CAAGTCTGCAAGTGAATACACC | CGTTGAAGGCACTATAAATGGG |
| *VvTAR* | ATTTTGACCTTCCAGCCTATGA | TAGTAAATGGCTTCACCTCAGG |
| *VvCYP79B1* | CTCTCACTTAGATCCTTCGGAC | CCCTCTGAAGTTGATAATCCGA |
| *VvYUC1* | TACAAGCACATTTTTCCACCAG | CGACAAGTAAAAGGCTTCAACA |
| *VvNIT1* | ACGGATTTCACTCCTGACTATG | TGTTCTCGAGACAGTCATTTGA |
| *VvAAO* | CACTACCATCCCCAAAATCTCT | CACAACTTCTTCTTCTTCTGGC |
